# Descriptions of long-term impact from inter-professional ethics communication in groups

**DOI:** 10.1177/09697330231160007

**Published:** 2023-03-15

**Authors:** Britt-Marie Wälivaara, Karin Zingmark, Catarina Fischer-Grönlund

**Affiliations:** 91874 Luleå University of Technology, Sweden; Umeå University, Sweden

**Keywords:** healthcare professional, inter-professional ethics communication, long-term impact, qualitative

## Abstract

**Background:**

On a daily basis, healthcare professionals deal with various ethical issues and it can be difficult to determine how to act best. Clinical ethics support (CES) has been developed to provide support for healthcare professionals dealing with complex ethical issues. A long-term perspective of participating in inter-professional dialogue and reflective-based CES sessions is seemingly sparse in the literature.

**Research aim:**

The aim was to describe experiences of impact of Inter-professional Ethics Communication in groups (IEC) based on Habermas’ theory of communicative actions, after 6 months from the perspective of an inter-professional team.

**Research design:**

A qualitative inductive approach was chosen, and individual interviews (*n* = 13) were conducted. Interview data were analysed using qualitative content analysis.

**Participants:**

The participants, 10 females and two males, represented assistant nurses, registered nurses, physicians, occupational therapists, physiotherapists, welfare officers and psychologists. Each had attended at least four IEC sessions.

**Ethical considerations:**

The study was approved by the Regional Ethical Review Board in Umeå, Sweden, and it has been undertaken in accordance with the Helsinki Declaration.

**Findings:**

Overall, the descriptions expressed a perceived achievement of a deepened and integrated ethical awareness that increased the participants’ awareness of ethically difficult situations as well as their own ethical thinking, actions and approaches in daily work. Perspectives were shared and the team become more welded. They carried the memories of the reflections within them, which was perceived as supportive when encountered new ethically situations.

**Discussion:**

Putting words to unarticulated thoughts may stimulate repeated reflections, leading to new insights and alternative thoughts.

**Conclusion:**

The outcome of IEC sessions 6 months following the last session can be described as an incorporated knowledge that enables actions in ethically difficult situations based on an ethical awareness both at a ‘We-level’ and an ‘I-level’.

## Introducion

On a daily basis, healthcare professionals deal with ethical issues related to decisions, priorities and other caring and medical situations,^1^ organisations and administrative systems.^2^ Daily practice involves interactions with patients, relatives and co-workers with different opinions, professional standpoints and values in particular situations.^3^ As a result, it can be difficult to determine how to act best,^4^ both at an individual level and as a professional team. The healthcare team comprises various professionals with different roles and perspectives of the care situations. Hence, a discrepancy of ethical reasoning among various healthcare professionals^5^ and further a lack of communication has been described.^6,7^ In a study by Silén, Svantesson and Ahlström,^8^ registered nurses (RNs) expressed a need for communicating ethical issues with physicians and called for a dialogue, both to better understand the physicians’ intentions and to convey their own aspects of the situation. It has also been found that RNs and physicians, in the same context and with similar ethical difficulties, shared feelings of uncertainty, loneliness and disempowerment when they were not able to communicate their struggles across professional boundaries.^9,10^ The lack of communication regarding ethically difficult care situations has been described as a feeling of becoming trapped in isolation, confusion, self-doubt and frustration.^10^

Clinical ethics support (CES), with various approaches has been developed in many countries to provide support for health professionals dealing with complex ethical issues.^11^ Clinical ethics consultations,^12^ and ethics committees,^13^ can, for example, provide advice, recommendations, and bases for decisions. Moral case deliberation (MCD)^14^ aims to promote the dialogue within the team by a collaborative, systematic reflection on clinical cases by using the dilemma method or Socratic dialogue.^11,15^ Various approaches are used to promote inter-professional dialogue and reflection on perceived ethical issues in daily practice, and consists of ethics rounds,^16^ ethics discussion groups,^17^ ethics reflection groups^18^ and a six step manual developed by the Centre of Medical Ethics (SME).^19^ MCD has been described as a ‘bottom-up’ perspective that might increase the possibilities for healthcare professionals to deal with ethical difficulties and daily decision-making.^11,20^ It has been suggested that MCD can work as an umbrella concept on the various approaches of inter-professional ethics dialogue.^21^ The CES in focus for this study entitled Inter-professional Ethics Communication (IEC) has an approach that is approximately in line with the ethics rounds. The organisational and communicative structure, however, is based on Habermas’ theory of a democratic dialogue.^22^ Habermas emphasises that moral principles are universal norms accepted by a common will. By communicative actions people may examine and validate norms and principles to be accepted as in the best interest for those it concerns. Prerequisites for communicative actions are an understanding-oriented dialogue approached by equity, openness, and review. Approach of equity means that everyone may feel free to communicate without any power claims.^23,24^ Openness means that participants have opportunities to express their views with statements that are truthful, valid, sincere and comprehensible. Review promotes a dialogue that continues until a common understanding among participants is achieved.^24^

The importance of providing dialogue and reflective-based ethics support has been stressed and impact,^25^ evaluations, outcomes^26,27^ and experiences of participation^28,29^ have been studied and described. Additionally, the communication process and communicative and organisational aspects of IEC have been studied.^23,30^ However, a more long-term perspective of participating in inter-professional dialogue and reflective-based ethics communication sessions is seemingly sparse in the literature. Knowledge is needed about IEC as an opportunity for healthcare professionals to understand and solve ethical problems at the moment. It is also important to gain knowledge if IEC can have a long-term impact where healthcare professionals gain knowledge and become equipped to face ethically difficult situations in everyday practice.

## Aim

The aim was to describe experiences of impact of inter-professional ethics communication in groups (IEC) based on Habermas’ theory of communicative actions, after 6 months from the perspective of an inter-professional team.

## Method

### Study design

In relation to the aim of the study, a qualitative, inductive approach was chosen in order to grasp the individual narrated experiences and perspectives.

### Context

This study was conducted at a ward for long-term rehabilitation providing care for people with extensive injuries who were in need of inter-professional rehabilitation. Since the inter-professional team often dealt with ethical issues, they applied for, and were offered regular IEC sessions, one-hour, every month during 1 year. These sessions were facilitated by external well experienced healthcare professionals with education in caring and medical ethics and trained in ethical support. The IEC concept was grounded in^24^ the notion of four prerequisites for a democratic dialogue: equality, possibility to examine, openness to other’s views and attitudes and a will to reach agreement or consensus. At the beginning of each session, one participant narrated a current, authentic care situation involving an ethical issue, and thereafter the participants reflected on the situation together. The conversation first focused on the participants’ feelings related to the situation, and then more specifically on the ethical issue and the value conflict involved. Questions were asked in order to identify the value conflict and commonly put it into words. The expressed intention with the communication process was to facilitate an agreement on how to understand, handle and resolve the value conflict.^22^

### Participants

Six months after the last IEC session, 13 participants of total 41 were invited to participate in a research interview. The criterion for the invitation was that the subject had participated in at least four of the IEC sessions. Ten female and three males agreed to be interviewed, that is, everyone who was invited, and there was professional representation from the whole inter-professional team. The participants consisted of assistant nurses, registered nurses, physicians, occupational therapists, physiotherapists, welfare officers and psychologists.^22^

### Data collection and analysis

In order to reach the individual deeper perspective, individual interviews were chosen. Interviews (*n* = 13) were conducted by the first author. The open-ended interviews took place in a private room in the hospital and lasted 27–58 min (mean = 42 min). An interview guide with question areas was used to ensure that the aim of the study was covered during the interviews. The initial question was *What have you learned by participation in IEC*, and the question areas were; *Experiences and impact of having participated in IEC, Impact on perception/way of thinking of ethics, Impact on facing ethical difficult situations and What is taken to clinic after IEC/learning impact.* The participants were asked to freely describe their experiences of IEC, and follow-up questions within the areas were used to stimulate the participants to share their experiences and provide a richer picture.

The interviews were digitally recorded, transcribed verbatim and analysed using qualitative content analysis inspired by Graneheim and Lundman.^31^ The entire text of the interviews was used in the analysis. The text was read through several times to obtain a sense of the whole and according to the aim, the text was divided into meaning units, which were condensed and assigned descriptive codes, which were close to the text. The codes were sorted in several steps, according to similarities and differences in content and at higher levels of abstraction, and later formulated into four categories. The latent message was interpreted and a theme was formulated. The analysis process was conducted and stored in NVivo 12 for Windows,^32^ which provides an audit trail from the original interview text during every step of the analysis to the categories and the theme, providing rigor during the analysis process. In this process the groups of text at different abstraction levels were compared with the original text to ensure trustworthiness in the analysis, and all authors took part in and discussed the analysis process to handle the pre-understanding. They had not been involved in the IEC sessions.

### Ethical considerations

Written and verbal information about the study was provided to each participant. It was made clear that participation was voluntary and that they could withdraw from the study at any time without explanation and without consequence. Confidentiality was assured, and it was explained that the findings would be presented confidentially. Before the interviews, all participants gave written informed consent. The interviewer was an extern and had not been involved in the IEC interventions. Approval to carry out the study was granted by the Regional Ethical Review Board in Umeå, Sweden (Dnr. 2012-338-31M) and it was undertaken in accordance with the Helsinki Declaration (2013).

## Findings

The findings show achievement of a deepened and integrated ethical awareness and are presented in one theme and four categories (Table 1). The content is visible in the text and supported in the citations.

**Table 1.** Descriptions of long-term impact of Inter-professional Ethics Communication (IEC) 6 months following the last sessions, theme (*n* = 1) and categories (*n* = 4).

Theme	Achievement of a deepened and integrated ethical awareness
Categories	We cracked our shells and became able to share perspectives
	We became more welded and learned to better cooperate and co-create
	I am carrying the memories of our reflections within me
	I have gained an increased comprehensive understanding of ethical issues

### Achievement of a deepened and integrated ethical awareness

The underlying meaning of the findings showed that the impact of IEC was hidden and not verbalised or reflected as new knowledge in the form of additional ethical principles or theories. However, the narrations revealed when sharing perspectives and reflecting on solutions ethical issues became visible and it was possible to reach enriching insights. The insights made an impression and influenced the understanding of ethical issues both professionally and privately. The narrations conveyed a message of having been part of an implicit learning or development process both from a ‘we’ and ‘I’ perspective with increased readiness for ethical situations, which can be understood as a deepened and integrated ethical awareness.

#### We cracked our shells and became able to share perspectives

The narratives provided a picture of the IEC sessions as a kind of sanctuary, where it was possible to crack one’s harder shell and open up to softer and more vulnerable personal dimensions. It was described as an opportunity to take part in colleagues’ different experiences and skills concerning, for example, meetings with reluctant patients. Issues were illuminated from several perspectives. To hear about other people’s emotions, thoughts and opinions was described as enriching. Insights were provided into issues that did not need to be labelled as right or wrong, but rather from different views and experiences. Opening up and sharing each other’s experiences as human beings and not just as professionals with different hierarchal positions, was described as an opportunity to show vulnerability while still being taken seriously.

*There is so much that is positive. It’s that…it opens up in some way. It becomes an open atmosphere. Everyone can say what they think, and in addition, everyone cares about what exactly that person thinks. Everyone thinks and everyone is allowed to think, and everyone can join the conversation. (Participant 12)*

*There we had the same…whatever profession, you have similar experiences. So that’s why you don’t need to…it wasn’t so important to say something yourself, you knew so well. But then, I spoke of my experiences too. (Participant 4)*

Participants compared their experiences from the IEC with other structured meetings they used to attend and described it as a complement to, for example, medical rounds, care planning, team rounds, debriefings, reflections, and coaching sessions/supervisions. They indicated that IEC sessions were unlike the other forums, because there was a specific focus on ethical issues, which was perceived as meaningful, since it made the ethical issues visible and gave space for existential reflections. Having been giving the opportunity to communicate and share frustrations related to ethically difficult care situations was stressed as important. Although, it had not been possible to find good or acceptable solutions for how to act in all of the caring situations, there was a sense of strength in sharing them. During the IEC period, a sense of consolation in not being alone was raised. The participants realised that they struggled with similar ethical issues and the sessions led to a more affirmative approach with each other.

*…and usually it’s so. If you take time and sit down and talk…if someone says something…and another says, “Well I also had the same experience!” But if you do not get the chance to sit down and talk about such pretty intimate and emotional stuff, then maybe it won’t come up either. Because when someone opens up, then it’s much easier for everyone else to do it. (Participant 9)*

*There might not be a solution either, but even so…you got to talk about it, and then everything was easier. Just that…I’m not alone in thinking this way, and how should I act now…and how should we do this? And then, there is not an answer for everyone, and we are all different and so on, but we have talked about this, and we know that the problem exists. It eases [the problem]. (Participant 12)*

#### We became more welded and learned to better cooperate and co-create

When recalling the IEC sessions, they described a sense of being a tighter, more cohesive team during the intervention. There were descriptions of a calmer atmosphere and climate within the group where staff members were more open and confirmatory to each other. IEC was described as promoting collaboration and strengthening generosity towards each other. Some participants put forward that the climate within the group was improved during the period with IEC.

*And then we kind of throw our balls away, and they bounce back with thoughts that we didn’t have, and then you get like… I hear what my colleagues say, and I can understand and relate to what they say. Yes, we both get to talk together and vent ourselves, and get this support from the leaders, with tools and feedback on what we do and how we think and what we feel. And so, you become both…calmer and more understanding and more collaborative as a working group. (Participant 8)*

Participants described that during the IEC period while discussing important ethical issues, they often reached agreement on how to act, co-operate and find a common, fair and clear approach in caring for patients and family members. There was conformity in the descriptions of promoted cooperation and co-creation during the IEC period among those who participated in the sessions. However, some participants did not experience increased cooperation at the unit as a whole and related that to the fact that not all staff members took part in the IEC. Others considered that the process affecting those involved in IEC led to a spreading effect changing the climate in the whole staff group. The opinions of the long-term effect varied. Some described that the ethical conversations continued within the professional team after IEC, and others described that they still find it difficult to discuss ethical issues with colleagues, especially if the colleague did not participate in IEC.

*It also affects you afterwards. It is easier, I think, to continue… You continue the discussion afterwards, and if you… We talk to each other as well in another way. (Participant 1)*

#### I am carrying the memories of our reflections within me

All participants were satisfied with the IEC. They voiced the feeling of being important and visible when they got the opportunity to participate, and they wanted to recommend to others that they take part. They expressed that the only things that could go wrong during IEC would be if participants did not respect each other or if the conversation got stuck in complaints instead of leading forward. During the IEC sessions, they could suggest what they wanted to discuss. A wide range of topics were suggested, including patient cases, complex care situations, ethical dilemmas, professional approaches to difficult patients and relatives, autonomy, integrity, setting boundaries, delivering bad news, honesty, challenging communications, personal attacks, drug problems, feedback and approaches to colleagues. The reflected dialogues were commented on as interesting and meaningful, and also helpful in the present. Participants broadened their arsenal for dealing with ethically difficult care situations and used their experiences from the IEC period as inspiration for how to reflect and act in current situations. This gave a sense of being better prepared for difficult situations in their professional role. There were also descriptions that IEC stimulated participants’ desire to learn more about ethics in their profession.

*But as you discuss a dilemma, or attitude or what it is, then I think I will use it for the next encounter or whenever it may be. But to say exactly what that is, I can’t do it. As it is with all knowledge, you have the understanding and the way to use it in encounters with patients or colleagues. (Participant 1)*

The notion of ethics in the everyday care context was described as difficult to grasp and could be understood as twofold; on the one hand, it is theoretical, but, on the other hand, it is something practical. Theoretical courses in ethics had been part of the participants’ education and internal training, and they did not consider the IEC sessions to increase their theoretical knowledge. They expressed that they learned something while participating in IEC but found it difficult to concretise exactly what; however, they indicated that IEC had specific usefulness in the clinic, and it affected their perceptions about how to apply ethics in practice.

*The concept of ethics is a bit difficult for me. In the past, I have seen it more as a theoretical…yes, quite a theoretical track, and now I think I can see it more on a practical, clinical level in some way. (Participant 1)*

#### I have gained an increased comprehensive understanding of ethical issues

Participants described that the dialogues during IEC had affected their readiness for meeting ethical challenges professionally at a deeper level and made them more aware of their reactions, thoughts and actions in caring situations. Concrete examples provided a greater understanding of patients’ and relatives’ different behaviours as well as colleagues’ actions. The dialogue described different ways of thinking in specific situations related to care and treatment, resulting in a greater awareness of morals and values.

*…a little bit, I think. I find it a little easier to understand it* [other people’s way of thinking]. *It has just turned around a little bit of my thinking and…yes, I have not only stuck to mine, my reactions. (Participant 4)*

In their narrations, the participants also described that IEC had affected them personally as they got the opportunity to reflect on their own ethical thinking, their actions and behaviour. Some participants stressed that the knowledge acquired during IEC was applied in private life, for example, in difficult situations related to care of elderly parents or in boundary situations with children. IEC was also described as supporting a broader understanding of earlier ethical issues in some of the participants’ private life. Others commented that IEC had not affected them personally.

*You do not distinguish between work and leisure; you are the same person. Well, I think it can, that you might…these discussions make you learn to take like one more step and turn things around. I think that I can…it is the same in the family as well as in private life. (Participant 7)*

*It’s, after all, it’s abstract things. It’s the question of ethics and morals and values and so on. So it’s not like saying that you have learned the multiplication table, but you do have maybe a bigger perspective, and more angles, and more tools, and a greater understanding. (Participants 8)*

## Discussion

IEC was described as a ‘cracking of shells’ and a possibility for the inter-professional team to share perspectives, become more welded and learn to better cooperate and co-create. The participants also described how they sometimes returned in memory to the reflective dialogues in the IEC sessions and had developed an increased readiness for ethically difficult situations.

It seems that the participants were not always aware of what they had learned. Hence, they expressed that they had not learned anything new after participating in IEC; however, in their ongoing narrations they conveyed new concepts or an in-depth understanding. During the analysis, it emerged that their theoretical knowledge in ethics had been translated into ethically difficult care situations during IEC, which could be interpreted as an increased ethical awareness and an in-depth knowledge. IEC promoted the potential of applying ethical knowledge and perceptions into practice. Everyday events and situations were viewed from an ethical perspective not reflected upon previously. This could be understood as in-depth knowledge of ethics and moral development.

In this study, the narrations presented a picture of the IEC sessions as a kind of sanctuary of trust, which enabled the participants to reach beyond the surface to ‘crack one’s shell’. Communicating ethical issues, sharing perspectives and emotions from both a professional and personal point of view opened up for extensive reflections. Participating in IEC enabled possibilities for the participants to express their point of views, listen to co-workers, be listened to and further they reached common new views. This is in line with Shotter,^33^ who suggested that trustful communication may lead to a common view in a deeper sense; hence, it opens us to a sensitivity to understand others involved and makes us aware of ourselves and our intentions. According to Koskinen and Lindström,^34^ there is a connection between narrating and listening. Narrating means that we share ourselves with others while listening to our innermost self. That, in turn, opens us up for new directions in life.^34^ Silfverberg^35^ refers to Hanna Arendt and considers that the picture of our personal self appears by talk actions in a community. Communicating and putting words to unarticulated thoughts may stimulate repeated reflections, leading to new and extended insights from which may emerge innovative and alternative thoughts.^35^

In this study, the communication entered in a common agreement for how to understand, act and approach situations of concern. In a study, Bartholdson et al.^36^ similarly described that professionals arrived at a comprehensive view of the situation by sharing experiences and perspectives. Even if everyone did not agree in all aspects, they still experienced team unification. Communication in IEC created a climate where the professionals became more open, generous, collaborative and confirmatory towards each other. Benhabib^37^ implied that people are different, have various experiences and, therefore, comprise diversity of aspects and viewpoints. By turning different perspectives and various experiences that complement each other into a reflexive dialogue, people may build the base for an extended mentality. This necessitates the ability to accept others’ perspectives.^37^

In this study, the reflected dialogues were viewed as meaningful and beneficial in the present and the experiences from the IEC period were used as inspiration for how to reflect and act in ethically difficult care situations. According to Biggs and Tang,^38^ reflective learning refers to an individual’s inner cognitive processes and an approach to what is considered meaningful learning. The participants understood ethics as twofold: on the one hand, it was theoretical, and on the other hand, it had practical implications. Martinsen,^39^ inspired by Løgstrup,^40^ warns of an abstract [theoretical] conceptual language, that is, laid down classifications, definitions and standards that might obstruct the important articulation of impressions that move us forward. Martinsen,^39^ describes concepts and uses Løgstrup’s phrase that the concept mill spews out abstract answers to its own questions, and once the concept use has started to grind, it is difficult to make it stop.

Martinsen and Kjerland^41^ highlight the importance of seeing with the heart’s eye, which means that the healthcare professional has to perceive and be touched when encountering the other. This requires an ability to empathise with the other and the other’s situation, which differs from conceptualising and manualising. During the reflected dialogue, the participants had the ability to see with the heart’s eye rather than focusing on ethical principles and theories. Hence, they expressed that their theoretical knowledge did not increase after participating in IEC sessions. However, IEC did affect how they applied ethics in practice. The reflected dialogues left memories within the participants, and they felt better prepared to deal with ethically difficult situations. This can be viewed as ‘incorporated knowledge’. Aristotle’s virtue *phronesis* concerns ethics, values and interests and refers to a situational and contextual judgment ability, which is acquired through practice and experience. The virtue *phronesis* presupposes an interaction between the general and the concrete, cf.^35^ This means that healthcare professionals need knowledge in applying general theoretical knowledge, such as ethical principles and theories, in concrete care situations. However, they also need to act based on ethical awareness, in relation to individual needs identified through the heart’s eye.

In the IEC sessions, not only perspectives of general knowledge but also individual relations in the concrete situation need to be identified through awareness and taken into consideration. Benhabib,^37^ has elaborated on Habermas discourse ethics theory and claims that if the ethical discourse only focuses by rational knowledge, ethical theories, principles and norms there is a risk for character and emotions to be neglected.^37^ According to Benhabib, the moral discourse originates in the everyday dialogue, to concern everyday moral caring situations, in relations to concrete others. A continuously ongoing ethical dialogue may lead to a development of ability to express one self, responsiveness for other persons and egalitarian reciprocity with a caring approach.^37^

In this study, the participants reflected on their approach to ethical thinking as increased awareness of reactions, thoughts, actions and experienced broadened understanding of various perspectives. Weidema et al.^42^ similarly found that nurses after participating in moral case deliberation (MCD) expressed improved awareness of other colleagues’ perspectives, broadened understandings and increased sensitivity to the moral dimensions of their work. In this study, the broadened understandings were expressed by compassion and a sensitivity for the patient’s and relative’s situations, which can be understood as development of ethical sensitivity. Ethical sensitivity was described by Rest and Narvaez,^43^ as a person’s empathy, ability to interpret and awareness of others’ perspectives. In a literature review by Lechasseur et al.,^44^ ethical sensitivity was further described as a person’s ability to use compassion and intelligence to recognise and interpret ethical tensions in relation to others. The ethical dialogue in this study revealed various perspectives of the situations and the participants experienced a comprehensive understanding. Van Der Zande, Baart and Vosman^45^ stated that ethical sensitivity enhances both explicit, expressive and implicit, tacit moral knowledge. Regular reflections with colleagues may discern and make both explicit and implicit moral knowledge visible. Caram et al.^46^ indicated that professional and moral dialogue may entail a source to learning and a recognition of oneself and others.

By sharing perspectives of the situation, the participants expressed moving beyond established attitudes of how people are expected to behave. The ethical communication opened to incorporate an understanding that people are different and there are variations of experiences and perspectives. Løgstrup,^40^ stated that we need to reflect on the ethical context beyond principles and norms in order to become open for other people’s understandings and what the situation demands from us. Overall, the descriptions expressed a perceived achievement of a deepened and integrated ethical awareness. IEC increased participants’ understanding and awareness of ethically difficult situations and dilemmas as well as an awareness of their own ethical thinking, actions and approaches in daily work.

## Methodological considerations

The interviewer had no previous relationship to the participants and the authors were not involved in the IEC sessions, despite this there was a pre-understanding that could affect the analysis process. To curb the pre-understanding, the authors discussed the analysis steps until consensus was reached, citation from raw data was given, and the results were also presented to a group of ethics experts, which strengthened the credibility of the analysis. The study was comprised of 13 participants, which can be considered a reasonable number, as the interviews were content-rich. It can be considered as a strength that a wide variety of healthcare professionals were represented in this study. The participants had attended at least four IEC sessions, however, several of them had attended more sessions, which can be a strength. It is unclear whether the result would have been affected if participants participated in fewer IEC sessions. It can be assumed that the research interview supported the participants to reflect on their own ethical awareness. We assume that the findings can be transferable to similar contexts. It is not clear what impact aspects such as the leader’s role and competence, group composition and ethical issues discussed may have on the outcome. This study shows participants’ experiences of impact of IEC sessions after 6 months. It is necessary to further study the perceptions after IEC sessions using longer term perspectives. In this study, healthcare professionals’ perspective was captured and in future studies it is necessary to also capture the perspective of patients’ and relatives’ when studying experiences of impact.

## Conclusion

The experiences of impact of IEC sessions can be described as a movement of knowledge from the brain, through the heart and out to the hands. This means that the tacit knowledge in the form of ethical principles and theories through dialogue with others is integrated and incorporated in one’s heart to create a deeper moral knowledge that enables action in ethically difficult situations based on an ethical awareness both at a ‘We’ level and an ‘I’ level.
